# Transforming growth factor-beta 1 produced by vascular smooth muscle cells predicts fibrosis in the gastrocnemius of patients with peripheral artery disease

**DOI:** 10.1186/s12967-016-0790-3

**Published:** 2016-02-04

**Authors:** Duy M. Ha, Lauren C. Carpenter, Panagiotis Koutakis, Stanley A. Swanson, Zhen Zhu, Mina Hanna, Holly K. DeSpiegelaere, Iraklis I. Pipinos, George P. Casale

**Affiliations:** Department of Surgery, University of Nebraska Medical Center, Omaha, NE USA; Department of Cellular and Integrative Physiology, University of Nebraska Medical Center, Omaha, NE USA; Department of Surgery and VA Research Service, VA Nebraska-Western Iowa Health Care System, Omaha, NE USA; 987690 Nebraska Medical Center, Omaha, NE 68198-7690 USA; 983280 Nebraska Medical Center, Omaha, NE 68198-7690 USA

**Keywords:** Peripheral artery disease, Skeletal muscle, Fibrosis, Transforming growth factor-beta 1, Vascular smooth muscle cells, Microvasculature

## Abstract

**Background:**

Lower leg ischemia, myopathy, and limb dysfunction are distinguishing features of peripheral artery disease (PAD). The myopathy of PAD is characterized by myofiber degeneration in association with extracellular matrix expansion, and increased expression of transforming growth factor-beta 1 (TGF-β1; a pro-fibrotic cytokine). In this study, we evaluated cellular expression of TGF-β1 in gastrocnemius of control (CTRL) and PAD patients and its relationship to deposited collagen, fibroblast accumulation and limb hemodynamics.

**Methods:**

Gastrocnemius biopsies were collected from PAD patients with claudication (PAD-II; N = 25) and tissue loss (PAD-IV; N = 20) and from CTRL patients (N = 20). TGF-β1 in slide-mounted specimens was labeled with fluorescent antibodies and analyzed by quantitative wide-field, fluorescence microscopy. We evaluated co-localization of TGF-β1 with vascular smooth muscle cells (SMC) (high molecular weight caldesmon), fibroblasts (TE-7 antigen), macrophages (CD163), T cells (CD3) and endothelial cells (CD31). Collagen was stained with Masson Trichrome and collagen density was determined by quantitative bright-field microscopy with multi-spectral imaging.

**Results:**

Collagen density increased from CTRL to PAD-II to PAD-IV specimens (all differences p < 0.05) and was prominent around microvessels. TGF-β1 expression increased with advancing disease (all differences p < 0.05), correlated with collagen density across all specimens (r = 0.864; p < 0.001), associated with fibroblast accumulation, and was observed exclusively in SMC. TGF-β1 expression inversely correlated with ankle-brachial index across PAD patients (r = −0.698; p < 0.001).

**Conclusions:**

Our findings support a progressive fibrosis in the gastrocnemius of PAD patients that is caused by elevated TGF-β1 production in the SMC of microvessels in response to tissue hypoxia.

**Electronic supplementary material:**

The online version of this article (doi:10.1186/s12967-016-0790-3) contains supplementary material, which is available to authorized users.

## Background

Lower leg ischemia, myopathy and limb dysfunction are distinguishing features of peripheral artery disease (PAD) caused by atherosclerotic blockages of the arteries supplying the legs. This disease affects over 27 million people in North America and Europe, where the prevalence is estimated to be 16 % of individuals 55 years and older, and is associated with a 5-year mortality rate of as high as 30 % [1–3]. Overall, about 11 million people with PAD are symptomatic [1, 4] and the majority experience claudication, i.e., walking-induced leg muscle pain that is relieved by rest (Fontaine Stage II). At later stages of the disease, PAD patients experience foot pain at rest (Fontaine Stage III) and non-healing ulcers, necrosis, and gangrene in the affected limb (Fontaine Stage IV). Hemodynamic changes were thought to be the sole cause of these symptoms [5–7]. However, we and others have demonstrated that a myopathy exists in the lower legs of PAD patients and contributes to limb dysfunction [8–13].

Myopathy in the affected legs of PAD patients is characterized by myofiber degeneration, elevated pro-fibrotic cytokines, and increased fibrosis [11, 14–18]. In a recent study, we found an average three-fold increase of transforming growth factor-beta 1 (TGF-β1; a potent inducer of fibrosis) in homogenates of gastrocnemius biopsies from PAD patients compared to controls. TGF-β1 was increased in the biopsies of all PAD patients, suggesting a chronic pro-fibrotic response [18]. Chronically elevated TGF-β1 is known to activate myofibroblasts to deposit extracellular matrix around the myofibers and associated microvessels which interferes with oxygen and nutrient delivery, producing myofiber degeneration [19–22]. Additionally, TGF-β1 can promote PAD myopathy by inducing myoblasts to differentiate into myofibroblasts rather than new myofibers [19–22].

TGF-β1 may be produced by immune cells and is known to induce pathological fibrosis [19, 20, 23], but other cells have also been reported to upregulate TGF-β1 expression and contribute to fibrosis [24–28]. During wound healing, TGF-β1 is released by immune cells to initiate the resolution phase of inflammation and to activate myofibroblasts to deposit extracellular matrix, of which a major component is collagen [29, 30]. It has been reported for various vascular diseases, including pulmonary arterial hypertension (PAH), that vascular smooth muscle cells (SMC) transition from a contractile to a synthetic phenotype that produces TGF-β1 [31–33]. In PAH, chronic hypoxia induces substantial thickening of vascular intima, media and adventitia caused by extensive proliferation and hypertrophy of vascular SMC that exhibit increased expression of TGF-β1, and deposition of extracellular matrix; features that extend to normally non-muscularized microvessels [34, 35]. In PAD, a similar transitioning of vascular SMC may occur in response to exercise-induced ischemia and the chronic intermittent hypoxia that are caused by atherosclerosis of the large vessels supplying the legs. If that is true, we expect this transitioning to also be related to the degree of hemodynamic compromise of the affected extremity and the clinical stage of PAD. In this study, we evaluated the cellular expression of TGF-β1 in the gastrocnemius of control and PAD patients with advancing Fontaine Stage, and its relationship to collagen deposition, fibroblast accumulation, and Ankle-Brachial Index (ABI).

## Methods

### Human subjects

The experimental protocol was approved by the Institutional Review Boards of the Veterans Affairs Nebraska-Western Iowa and University of Nebraska Medical Centers. All subjects gave informed consent.

#### PAD groups

We recruited 25 patients with claudication (Fontaine Stage II, PAD-II) and 20 patients presenting with tissue loss (Fontaine Stage IV, PAD-IV) who were undergoing lower extremity operations for symptomatic PAD. The diagnosis for each PAD patient was established on the basis of medical history, physical examination, decreased ankle brachial index (ABI < 0.9), and computerized or standard arteriography that revealed stenotic and/or occluded arteries supplying the lower extremities. PAD-II patients presented with intermittent claudication, but no rest pain or tissue loss. PAD-IV patients presented with non-healing ulcers and/or gangrene.

#### Control group

We recruited 20 control patients (CTRL) who were undergoing lower extremity operations for indications other than PAD. Control patients led sedentary lifestyles and had no history of PAD symptoms. CTRL patients had normal blood flow to their lower limbs as indicated by normal lower extremity pulses at examination and normal ABI at rest and after stress.

#### Biopsy

Gastrocnemius samples weighing approximately 250 mg were obtained from the anteromedial aspect of the muscle belly, 10 cm distal to the tibial tuberosity. All biopsies were obtained with a 6 mm Bergstrom needle. Some samples were frozen in liquid nitrogen for biochemical analysis while others were placed immediately into cold methacarn. After 48 h in methacarn, the specimens were transferred to cold 50 % ethanol, and subsequently embedded in paraffin.

### Quantitative assessment of collagen

#### Masson Trichrome stain

Paraffin-embedded biopsies sectioned at 4 microns were stained with a Masson Trichrome Kit according to manufacturer’s protocol (Fisher Scientific Richard Allan Scientific #22-110-648, Pittsburg, PA, USA). Briefly, slide specimens were deparaffinized in xylene, hydrated and then incubated at room temperature in Bouin’s fixative for 16 h. Nuclei were stained with Weigert’s Hematoxylin, myofiber cytoplasm with Scarlet Red, and collagen with Aniline Blue dye, with washes in-between steps. Stained slide specimens were dehydrated and mounted with Permount (Fisher Scientific #SP15-100, Pittsburg, PA, USA).

#### Image acquisition and spectral analysis

Microscopic fields of the five most fibrotic regions were collected from each Masson Trichrome stained slide (20× objective). Muscle tissue completely filled each chosen microscopic field. Imaging was implemented by multispectral wide-field microscopy, using a Leica microscope (North Central Instruments DMRXA2 Model, Plymouth, MN, USA) coupled with the Nuance EX Multispectral Imaging System (PerkinElmer N-MSI-EX Model, Waltham, MA, USA) that incorporates a CCD camera and liquid crystal tunable filter. This system generates an absorbance spectrum at each pixel of a two-dimensional spatial image of the specimen. The Nuance software quantitatively extracts the grey scale image of deposited collagen, which is then transferred to the Image-Pro^®^ Plus image analysis software (Media Cybernetics, Warrendale, PA, USA) for quantification of collagen area and density. Collagen density was determined as area-weighted mean pixel intensity (12-bit grey scale). Quantitative analysis was performed blinded from the group status.

#### Validation of spectral analysis

Spectral analysis was validated by the hydroxyproline assay for collagen content in whole muscle. Total collagen content of human gastrocnemius specimens was measured by a hydroxyproline assay based on a previously established protocol for skeletal muscle [36]. To determine intersession reliability, slide specimens of biopsy samples from ten patients were stained by Masson’s Trichrome in two separate analytical sessions and assessed by spectral analysis. Detailed methodologies and validation results can be found in “Additional file 1”.

### Quantitative fluorescence microscopy

We describe a novel application of quantitative fluorescence microscopy (QFM), a technique developed by our laboratory that has previously been validated using multiple biomarkers of PAD and other diseases, such as prostate cancer [17, 37–41].

#### Quantitative measurement of TGF-β1 expression

Duplicate slide specimens of gastrocnemius biopsies were exposed to a rabbit anti-TGF-β1 antibody (Abcam Ab53169, Cambridge, MA, USA) and a mouse anti-CD31 antibody (Abcam Ab9498, Cambridge, MA, USA; to validate identification of microvessels). Slides were then treated with both goat anti-rabbit IgG secondary antibody coupled with Alexa Fluor^®^ 555 and goat anti-mouse IgG secondary antibody conjugated with Alexa Fluor^®^ 488 (Life Technologies #A21429 and #A11029, Carlsbad, CA, USA). Isotype control slides were treated with rabbit IgG (Vector Laboratories #I-1000, Burlingame, CA, USA) and mouse IgG1 (eBioscience #14-4714-85, San Diego, CA, USA) at the same concentration as the anti-TGF-β1 and anti-CD31 antibodies, respectively. For epitope recovery, Tris buffer (pH 9.0) was used. All labeling procedures were performed with a fully programmable, robotic autostainer (BioGenex i6000 Model, Fremont, CA, USA). The labeled specimens were mounted in ProLong Gold^®^ anti-fade medium containing 4’,6-Diamidino-2-phenylindole (DAPI; a nuclear stain) (Life Technologies #P36931, Carlsbad, CA, USA). Fluorescence images were captured with a Leica epifluorescence wide-field microscope (10× objective) (North Central Instruments, DMRXA2 Model, Plymouth, MN, USA) and CCD camera (Hamamatsu Photonics, Orca C4742 Model, Bridgewater, NJ, USA), with Hamamatsu software (HCImage 4.0). All fields of each specimen are captured and a montage of the gray scale images was generated for analysis with Image-Pro^®^ Plus (Media Cybernetics, Bethesda, MD, USA). TGF-β1 positive events in the microvasculature are partitioned and both event area and mean pixel intensity are determined for each (12-bit grey scale). The sum of the products of area and mean pixel intensity of all positive events per microscopic field was computed and normalized to the total area of muscle tissue specimen analyzed. Quantitative analysis was performed blinded from the group status.

#### Validation of quantitative fluorescence microscopy

QFM measurements of TGF-β1 were validated by comparing results with ELISA and qPCR measurements of muscle homogenates from PAD-II and CTRL patients (N = 13 in each group). TGF-β1 protein expression was measured as part of a customized Human Inflammatory Cytokines Multi-Analyte ELISArray Kit (Qiagen, Valencia, CA, USA).

To measure TGF-β1 gene transcripts in skeletal muscle biopsies, RNA extraction, reverse transcription reactions, and qPCR were performed as previously described [40, 42] and levels were normalized to myosin gene transcripts. Intrasession reliability was determined by comparing the TGF-β1 measurement of each slide from the mean of its duplicate pair. Intersession reliability was determined from the averages of patient biopsy specimens, analyzed a second time in the next analytical session. Detailed methodologies and validation results can be found in “Additional file 1”.

### Co-localization of TGF-β1 and Ki-67 to candidate cells by immunofluorescence

Individual slides were treated with primary anti-TGF-β1 antibody (Abcam Ab53169, Cambridge, MA, USA) and primary antibody specific for each of the following cell types. For endothelia, we used an antibody against CD31 (1:50 dilution) (Abcam Ab9498, Cambridge, MA, USA); for vascular smooth muscle cells, an antibody against high molecular weight caldesmon (1 ug/mL) (Abcam Ab1826, Cambridge, MA, USA); for fibroblasts, an antibody for TE-7 (1:10 dilution) (Millipore CBL271, Billerica, MA, USA); for macrophages, antibodies for CD163 (2.5 ug/mL) (Abcam Ab156769, Cambridge MA, USA) and CD68 (1 ug/mL) (Fisher Scientific Thermo MS-397, Pittsburg, PA, USA); for T cells, an antibody for CD3 (1ug/mL) (Abcam Ab699, Cambridge, MA, USA). To detect proliferative cells, we labeled with an antibody against Ki-67 (5 ug/mL) (Abcam Ab15580, Cambridge, MA, USA) and ProLong Gold^®^ anti-fade medium with DAPI nuclear stain (Life Technologies #P36931, Carlsbad, CA, USA). For all labels, we used Tris buffer (pH 9.0) for epitope recovery, except for Ki-67 where we used citrate buffer (pH 6.0). Isotype control slides were stained with rabbit IgG (Vector Laboratories #I-1000, Burlingame, CA, USA) or mouse IgG1 (eBioscience #14-4714-85, San Diego, CA, USA), IgG2a (eBioscience #14-4724-85, San Diego, CA, USA), or IgG2b (eBioscience #14-4732-85, San Diego, CA, USA) at the same concentration as their respective primary antibodies. Primary antibodies to TGF-β1 and Ki-67 were labeled with a goat anti-rabbit IgG secondary antibody coupled with Alexa Fluor^®^ 555 (Life Technologies #A21429, Carlsbad, CA, USA) and all other primary antibodies were labeled with a goat anti-mouse IgG secondary antibody coupled with Alexa Fluor^®^ 647 (Life Technologies #A21236, Carlsbad, CA, USA). All labeling procedures were performed with a fully programmable, robotic autostainer (BioGenex i6000 Model, Fremont, CA, USA).

### Detection of fibroblast accumulation by immunohistochemistry

Duplicate slide specimens were deparaffinized and heated in Tris buffer (pH 9.0) for epitope recovery. Specimens were blocked with 10 % goat serum for 5 min, treated with primary antibody against TE-7 (1:10 dilution) (Millipore CBL271, Billerica, MA, USA), a highly specific marker of fibroblasts [43–45], and then incubated overnight for 14 h. An isotype control treated with the same concentration of mouse IgG1 (EBioscience #14-4714-85, San Diego, CA, USA) was included with each duplicate antibody-treated slide. Specimens were treated with peroxidase-conjugated secondary antibody according to instructions from the DAB Polink 2 Kit (GBI Labs #D22-60D, Bothell, WA, USA). Hematoxylin (Fisher Scientific Richard Allan Scientific #72511, Pittsburg, PA, USA) was used as the counterstain for 4 min. Slides were dehydrated and mounted in Permount (Fisher Scientific #SP15-100, Pittsburg, PA, USA). Staining was implemented with a fully programmable, robotic autostainer (BioGenex i6000 Model, Fremont, CA, USA).

### Statistical analyses

The baseline characteristics between PAD and controls subjects were compared using general linear models for continuous variables and Chi square tests for categorical variables to determine confounders. Confounding variables were covariates in subsequent analyses. For all biological parameters, group differences were determined by analysis of covariance (ANCOVA) and evaluated post-hoc by Bonferroni adjusted t tests. Correlations were assessed by the Pearson test. All statistical analyses were performed with SPSS 20 (IBM, Armonk, NY, USA) using a confidence level of 95 %.

## Results

### Patient demographics

Data for CTRL, PAD-II, and PAD-IV patients are presented in Table 1. On average, PAD-IV patients were 6 years older than PAD-II (p = 0.047) and 8 years older than CTRL (p = 0.006) patients. More PAD-IV patients had diabetes than PAD-II and CTRL patients (χ^2^ = 5.55, p = 0.006; both p < 0.05). Age and diabetes were treated as covariates in all subsequent analyses.

**Table 1:** Demographics of study groups

	CTRL	PAD-II	PAD-IV	p value
Number of patients	20	25	20	N/A
Mean age (years)	62.1 ± 5.40	64.1 ± 7.80	69.9 ± 9.40*	0.011
Gender (male/female)	19/1	24/1	20/0	0.983
Height (m)	1.75 ± 0.07	1.76 ± 0.06	1.77 ± 0.06	0.576
Weight (kg)	91.0 ± 17.4	87.5 ± 18.8	85.0 ± 21.1	0.598
Body mass index	29.9 ± 6.40	28.2 ± 5.16	28.4 ± 6.61	0.593
Obesity^a^ (%)	45	36	25	0.416
Smoking (%)	50.0	56.0	30.0	0.202
Diabetes Mellitus (%)	25.0	20.0	65.0*	0.004
Dyslipidemia (%)	55.0	80.0	60.0	0.166
Coronary artery disease (%)	20.0	36.0	35.0	0.701
Myocardial infraction (%)	0.00	8.00	0.00	0.192
Hypertension (%)	65.0	84.0	90.0	0.116
Statins Medication (%)	70.0	84.0	65.0	0.317
Renal insufficiency^b^ (%)	5.00	12.0	20.0	0.352
Ankle Brachial Index^c^ (minimum–maximum)	1.04 ± 0.11* (0.79–1.20)	0.55 ± 0.22* (0.10–0.95)	0.22 ± 0.13* (0.00–0.44)	<0.001

*CTRL* Control subject, *PAD*-*II* PAD patient at Fontaine Stage II, *PAD*-*IV* PAD patient at Fontaine Stage IV

* p < 0.05 compared to each of the other two groups by post-hoc Bonferroni adjusted t tests

^a^Obesity: Body mass index >30

^b^Renal insufficiency: Creatinine clearance <60 ml/min/1.73m2

^c^ABI: data presented as mean ± standard deviation

### PAD gastrocnemius specimens exhibited increased collagen deposition with advancing disease stage

**Fig. 1:**
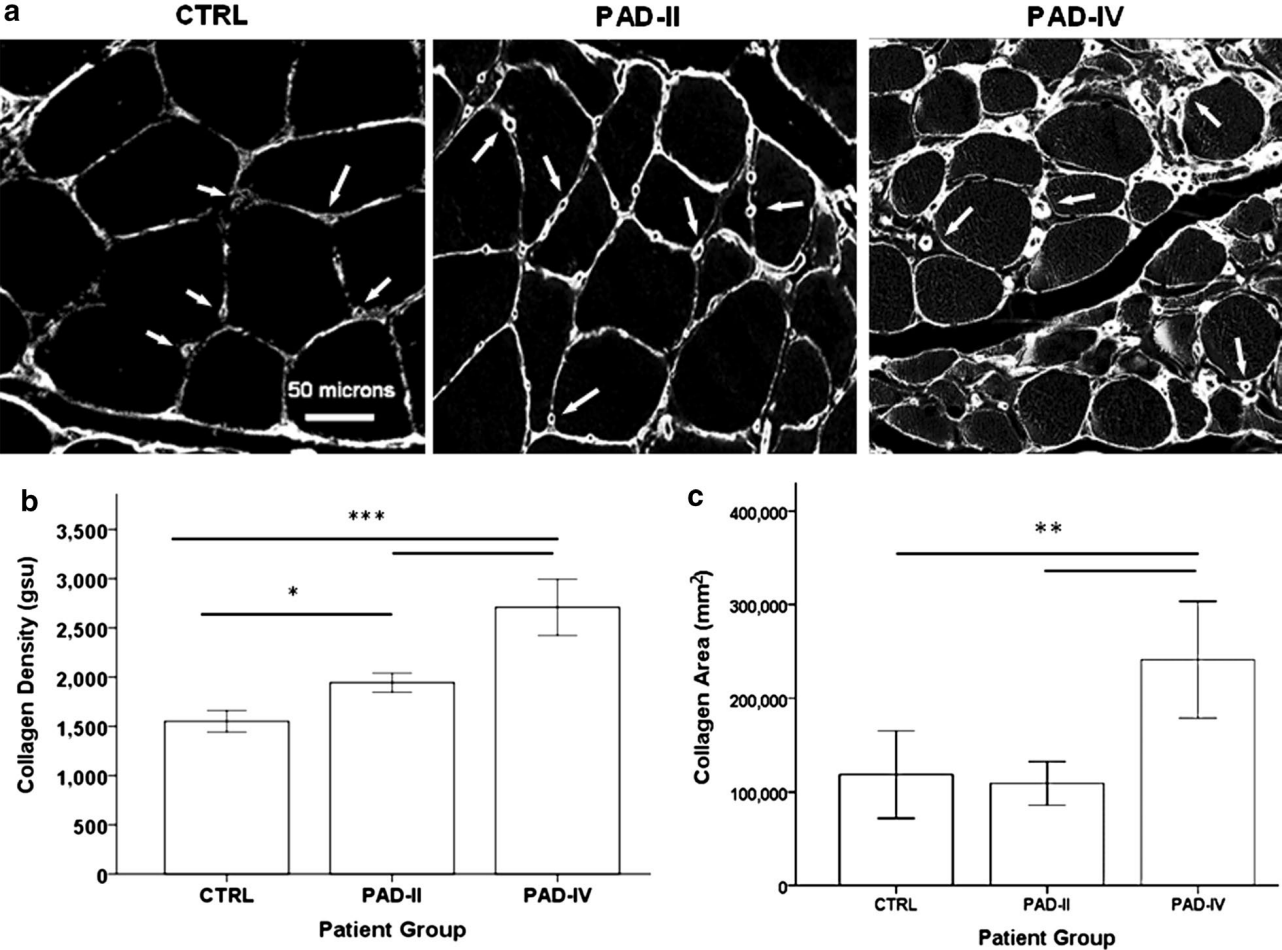
Collagen deposition in the gastrocnemius of CTRL and PAD patients with claudication and tissue loss. **a** Representative greyscale images of gastrocnemius specimens stained with Masson Trichrome were captured by multi-spectral, bright-field microscopy (20× objective). Specimens were collected from control subjects (CTRL) and PAD patients at Fontaine Stage II (claudication, PAD-II) and Stage IV (tissue loss, PAD-IV) disease. Myofibers delineated by collagen staining, appear black. Collagen density and area are represented by the intensity and extent of the *bright pixels*, respectively. *Arrows* point to collagen deposition associated with microvessels. **b** Collagen density and **c** collagen area in specimens of CTRL (n = 20), PAD-II (n = 25), and PAD-IV (n = 20) gastrocnemius were analyzed by quantitative multi-spectral microscopy. Collagen density was calculated as area-weighted mean intensity of all collagen events per specimen. Data are presented as mean ± standard error of the mean. Significance denoted as *p < 0.05; **p < 0.01; ***p < 0.001

Spectral imaging revealed increased collagen density with higher Fontaine stage, which became diffuse in Stage IV muscle (Fig. 1a). Collagen density and area in PAD muscle was increased between the myofibers and around the lumen of microvessels. The most noticeable pathological change was dense collagenous investment of the microvessels of PAD muscle (arrows). Spectral analysis established increased collagen density (p < 0.001) and area (p < 0.001) at the higher Fontaine stage. Collagen density in the PAD-IV patients (2708.8 ± 612.3 gsu) was 40 and 75 % greater than in PAD-II and CTRL patients, respectively (1969.9 ± 277.5 and 1551.7 ± 232.8 gsu; both p < 0.001), while collagen density was 25 % greater in PAD-II compared to CTRL (p = 0.015) (Fig. 1b). Collagen area was approximately twice as great in PAD-IV (241,179 ± 133,159 mm^2^) compared to either PAD-II or CTRL gastrocnemius (109,179 ± 56,481 mm^2^ and 118,624 ± 99,559 mm^2^; both p < 0.01), with no difference between PAD-II and CTRL (Fig. 1c). These data suggest that increased collagen deposition occurs first around microvessels and then expands throughout the extracellular matrix between myofibers and myofascicles as PAD advances.

### TGF-β1 expression is tightly linked to myofibrosis of PAD gastrocnemius

**Fig. 2:**
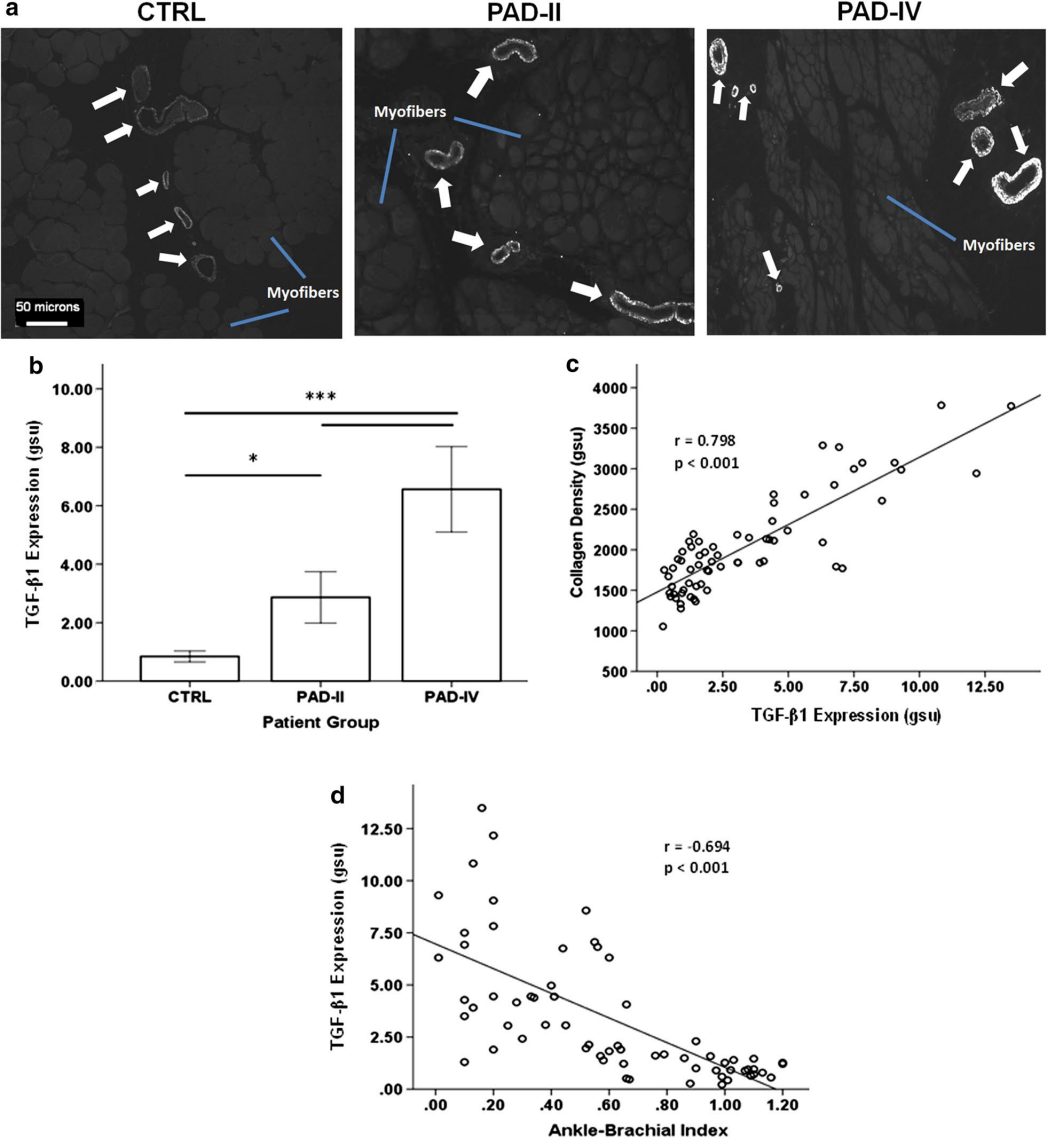
TGF-β1 expression in CTRL and PAD gastrocnemius and its relationship with collagen density and ABI. **a** Representative greyscale images of gastrocnemius microvessels positive for TGF-β1 labeling (*arrows*) were captured with a wide-field fluorescence microscope (10× objective). Unlabeled myofibers (*blue line*) appear *grey* against *black* background. Specimens collected from control subjects (CTRL) and PAD patients at Fontaine Stage II (claudication, PAD-II) and Stage IV (tissue loss, PAD-IV) disease were labeled with primary antibody specific for TGF-β1 and a fluorescent secondary antibody. Both the intensity and extent of TGF-β1 labeling were increased in the microvessels of PAD versus CTRL and PAD-IV versus PAD-II specimens. **b** TGF-β1 expression was determined by quantitative fluorescence microscopy and defined as the sum of the products of area and mean pixel intensity of all positive events per microscopic field, normalized to the total area of specimen in the same field. The relationships between normalized TGF-β1 expression and collagen density (**c**), and Ankle-Brachial Index (**d**) were determined by the Pearson correlation analysis. Data are presented as mean ± standard error of the mean and significance is denoted as *p < 0.05; **p < 0.01; ***p < 0.001

QFM imaging localized TGF-β1 expression to the vasculature of both CTRL and PAD muscle, with no detectable labeling outside of the vascular walls (Fig. 2a). TGF-β1 expression was uniformly low in CTRL muscle and exhibited a progressive increase in PAD-II and PAD-IV muscle. PAD-IV gastrocnemius had approximately 2.5- and 8-fold greater expression of TGF-β1 compared to PAD-II and CTRL patients (6.56 ± 3.12 vs. 2.89 ± 2.12 and 0.842 ± 0.399 gsu, respectively; both p < 0.001), while PAD-II had 3.5-fold more TGF-β1 than CTRL (p < 0.05; Fig. 2b). Vascular TGF-β1 expression positively correlated with collagen density across all subjects (N = 65) in this study (*r* = 0.798, p < 0.001; Fig. 2c). Separate analysis of the PAD patients alone (PAD-II and PAD-IV; N = 45) and the CTRL subjects alone (N = 20) revealed a significant correlation between TGF-β1 and collagen density in the PAD group (*r* = 0.854, p < 0.001), but not in the CTRL group (*r* = 0.119, p = 0.618), indicating that the pathological fibrosis in PAD is driven by TGF-β1 expression. Across all subjects (N = 65) in this study, vascular TGF-β1 expression increased with decreasing ABI (r = −0.694, p < 0.001; Fig. 2d), which is an indicator of blood flow (hemodynamic) compromise and increased ischemia of the lower limbs. The relationship between TGF-β1 and ABI remained significant when analyzing PAD patients alone (*r* = −0.543, p < 0.001). These findings identify increased microvascular TGF-β1 expression as a characteristic of PAD muscle and establish an association between increased TGF-β1 expression and muscle fibrosis and between increased TGF-β1 expression and advancing disease stage. Additionally, they show that as the blood flow (and presumably oxygenation) to the leg is decreased by atherosclerotic blockages in the arteries supplying the legs (reflected by decreasing ABI) there is a corresponding increase in the expression of TGF-β1 expression in the microvessels of the muscles of the affected leg.

### Increased expression of TGF-β1 is associated with accumulation of fibroblasts and collagen deposition in PAD gastrocnemius

**Fig. 3:**
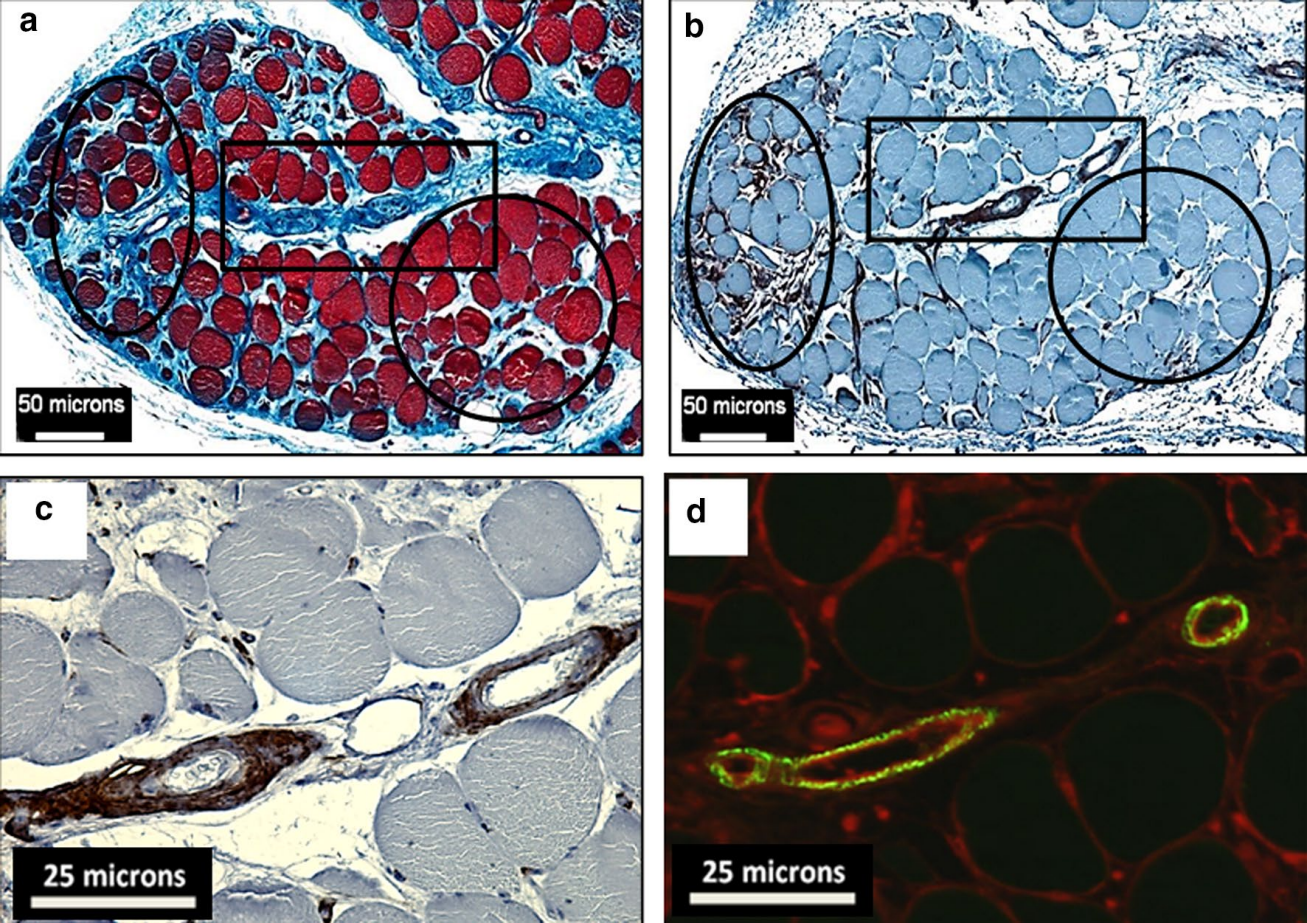
Association of TGF-β1 expression with fibroblast accumulation and collagen deposition in gastrocnemius of PAD patients. All images are of the gastrocnemius of a representative PAD patient who presented with tissue loss. **a** Masson Trichrome staining reveals highly fibrotic regions (*blue labeling*) around myofibers (*oval*) and microvessels (*rectangle*), and a region with relatively little fibrosis (*circle*). **b** A neighboring 4-micron section was labeled by immunohistochemistry with anti-TE-7 antibody, a fibroblast marker which identified fibroblasts in the same three regions of interest. **c** High magnification of the rectangular region of interest reveals the extent of fibroblast accumulation with microvessels. **d** A neighboring section labeled by immunofluorescence for TGF-β1 (*green* *fluorescence*) shows the intensity and extent of TGF-β1 labeling of microvessels within the *rectangular region* of interest. Wheat Germ Agglutinin (WGA; *red fluorescence*) labeled membranes and was used to delineate myofibers

To further evaluate the concept of a TGF-β1 dependent myofibrosis in PAD, we selected three adjacent gastrocnemius sections and stained the first section for collagen with Masson Trichrome, the second for TE-7 positive fibroblasts by immunohistochemistry, and the third for TGF-β1 by immunofluorescence. Images from a PAD-IV patient with significant fibrosis are shown in Fig. 3. In areas of dense collagen deposition (ellipse, Fig. 3a), we observed a high density of fibroblasts (ellipse, Fig. 3b). This is in contrast to areas containing relatively little collagen (circle, Fig. 3a), which had very few fibroblasts (circle, Fig. 3b). Vessels near the heavily fibrotic areas (high magnification of the square in Fig. 3a) contain a high density of fibroblasts (Fig. 3c) in association with intense TGF-β1 labeling (Fig. 3d) and dense adventitial collagen (square, Fig. 3a). These observations are consistent with the pro-fibrotic activity of TGF-β1, where TGF-β1 activates motile fibroblasts that deposit collagen (29, 30), and establish a spatial association of increased TGF-β1 expression, fibroblast accumulation, and collagen deposition as a characteristic of PAD pathophysiology. They also further demonstrate that the microvessels of ischemic legs play a key role in the pathophysiology of PAD myofibrosis.

### TGF-β1 is expressed by vascular smooth muscle cells in the microvessels of PAD gastrocnemius

**Fig. 4:**
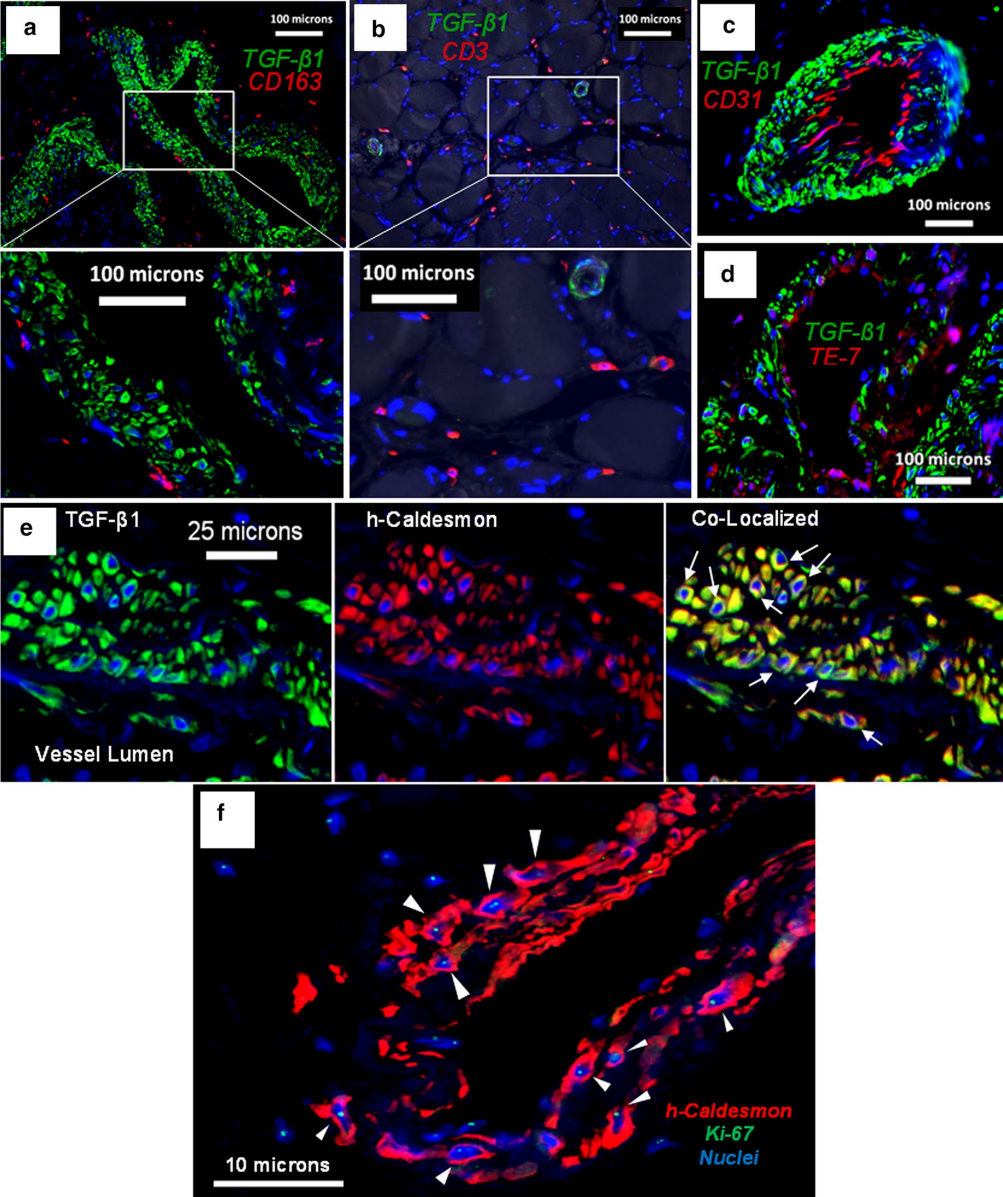
Evaluation of candidate vascular cells for TGF-β1 expression. All immunofluorescence images are from PAD patients who present with tissue loss and are representative of all diseased patients in our study. **a** TGF-β1 labeling (*green*) does not co-localize with CD163 positive macrophages (*red*) present at relatively high density in the adventitia of microvessels. High magnification of the boxed region reveals cellular labeling of CD163 around DAPI stained nuclei (*blue*). **b** TGF-β1 labeling (*green*) does not co-localize with CD3 positive T cells (*red*) that are located typically around myofibers near microvessels. High magnification of the *boxed region* reveals cellular labeling of CD3 around DAPI stained nuclei (*blue*). **c** TGF-β1 labeling (*green*) does not co-localize with CD31 positive endothelial cells (*red*) that are characteristically located in the intima of microvessels. **d** TGF-β1 labeling (*green*) does not co-localize with TE-7 positive fibroblasts (*red*). **e** TGF-β1 labeling (*green*) co-localizes with high molecular weight caldesmon (h-Caldesmon) a specific marker of smooth muscle cells (*red*). *Arrows* point to rhomboidal morphology characteristic of secretory SMC. **f** The proliferation marker Ki-67 (*green*) is expressed in nuclei (*blue*) of h-Caldesmon positive SMC (*red*) that highly express TGF-β1 as determined with a neighboring tissue section

To determine the specific cellular source of TGF-β1 expression in the microvessels of PAD gastrocnemius, we implemented co-localization studies with antibodies for highly specific markers of candidate vascular cells, including endothelial cells, SMC, fibroblasts, macrophages, and T cells (Fig. 4). TGF-β1 labeling did not co-localize with CD163 positive macrophages (Fig. 4a), CD3 positive T cells (Fig. 4b), or CD31 positive endothelia (Fig. 4c). TE-7 positive fibroblasts located in the adventitia were not positive for TGF-β1, nor were cells stained with TE-7 in the intima, which may represent endothelial-mesenchymal transition (Fig. 4d). TGF-β1 co-localized with cells expressing high molecular weight caldesmon, a marker of SMC that is not found in macrophages or fibroblasts [46, 47] (Fig. 4e). Prominent co-localization was observed in rhomboidal-shaped SMC located in the subendothelial region (arrows). This has been reported for other vascular fibrotic pathologies, where SMC are activated toward the pro-fibrotic synthetic phenotype, including in atherosclerotic arterial plaques. Many of the rhomboidal SMC stained positive for Ki-67 in the nuclei (arrowheads; Fig. 4f), indicating they are proliferative and activated. The finding that TGF-β1 is derived from locally proliferative SMC rather than immune cells in PAD myofibrosis is novel.

## Discussion

This study established that vascular TGF-β1 is strongly associated with myofibrosis during the progression of PAD. Using rigorous quantitative methods, we have shown that vascular expression of TGF-β1 and collagen deposition in PAD gastrocnemius increased in parallel with advancing disease severity. Qualitatively, TGF-β1 production was limited to cells in the walls of the microvessels in affected PAD muscle. In advanced disease, these cells expressing TGF-β1 were present at high density and were associated with accumulation of fibroblasts and increased deposition of collagen around the vessels and throughout an expanded interstitium. Our findings suggest that increased vascular TGF-β1 induces PAD myofibrosis by activating fibroblasts that proliferate and either stay locally around the microvessels or migrate between myofibers and around myofascicles into areas of myofiber degeneration, thus being responsible for the perivascular and endomysial/perimysial fibrosis we and others [48, 49] have shown in the affected leg muscles of PAD patients. Accumulation of fibrous tissue in the PAD muscle can affect the muscle both by compromising the function of its myofibers and myofascicles but also by interfering with the function of its microvessels. The fibrosis of microvessels in PAD muscle has been shown by us and others [48, 49] to involve both collagenous thickening of the capillaries and muscularization and fibrosis of normally non-muscularized microvessels and is known to impair diffusion of gases and small molecules between the vasculature and end organ parenchyma, probably enhancing the tissue hypoxia and inducing myofiber degeneration in the affected PAD legs. TGF-β1 may also be contributing to PAD myopathy by suppressing skeletal muscle regeneration by inducing myoblasts to differentiate into myofibroblasts rather than new myofibers [21, 22]. This model of PAD myofibrosis is consistent with the contribution of TGF-β1 to fibrosis and muscle degeneration seen in other skeletal myopathies [19] and myocardiopathies [50, 51].

Another novel finding of this study is that TGF-β1 is expressed exclusively by vascular SMC in PAD muscle. Our co-localization studies show conclusively that TGF-β1 is produced by vascular SMC and not in the associated fibroblasts, suggesting that these fibroblasts are activated by TGF-β1 produced in the SMC. These observations again point to a widely reported mechanism of fibrosis in human fibrotic diseases, in which TGF-β1 activates fibroblasts to myofibroblasts. Our co-localization studies also revealed that macrophages and T cells, which are reported to be the source of TGF-β1 in other skeletal myofibroses, did not express detectable TGF-β1 in PAD muscle [19, 20]. This is likely due to the different etiology of PAD myofibrosis compared to other skeletal myopathies, of which the muscular dystrophies are the best studied. The primary cause of PAD myopathy is atherosclerotic stenoses and occlusions of large arteries that supply the legs, which produce ischemia and hypoxia in the lower extremities [4]. Over time, chronic hypoxia may damage the muscle myofibers to produce their characteristic degeneration and induce SMC of the microvessels to increase TGF-β1 expression and cause myofibrosis. In contrast, muscular dystrophies originate from mutated genes that code for defective skeletal muscle proteins primarily involved in transmitting sarcomeric forces to the extracellular matrix [19, 20]. In muscular dystrophies, repeated cycles of myofiber degeneration and regeneration induce a chronic inflammatory response that includes TGF-β1 production by activated immune cells and produces the myofibrosis. Progressive fibrosis in PAD muscle appears not to be a response to chronic inflammation. We have profiled cytokines in the gastrocnemius of moderately diseased PAD patients and, indeed, did not find a generalized inflammatory signature [18]. The unique cytokine milieu produced by chronic ischemia in PAD gastrocnemius may explain why we did not observe detectable TGF-β1 in macrophages and T cells as is seen, e.g., in muscular dystrophies. Overall, the vascular etiology of PAD myofibrosis points to the possibility that chronic hypoxia of microvessels causes increased expression of TGF-β1 by SMC.

In PAD, hypoxic injury may cause transitioning of SMC from a contractile to a synthetic phenotype that is pro-fibrotic. Vascular SMC are normally contractile, with a spindle-shaped morphology and an abundance of contractile proteins that allow them to regulate vessel diameter. Under various pathological conditions marked by hypoxia, SMC can acquire a synthetic phenotype marked by rhomboidal morphology, decreased contractile proteins, and the ability to proliferate, migrate, and deposit collagen [31, 32]. In this study, we found that patients with lower ABI had greater expression of TGF-β1 across Fontaine Stages of disease, which suggests that hypoxia induces SMC to increase TGF-β1 expression. This is supported by our observation that many SMC with increased TGF-β1 expression were proliferative and exhibited a rhomboidal morphology that is characteristic of synthetic SMC.

The responses of vascular SMC to hypoxia in the ischemic muscle of PAD patients are likely similar to those in pulmonary arterial hypertension (PAH) and may provide insight into the mechanisms by which TGF-β1 expression is increased in PAD muscle. PAH is a condition characterized by substantial thickening of the vascular wall caused by extensive proliferation of SMC that exhibit increased TGF-β1 expression and deposition of collagen [34, 35]. Hypoxia alone can stimulate proliferation of human pulmonary SMC in culture [34]. Alternatively, growth factors released by cultured endothelial cells exposed to hypoxia also can stimulate proliferation of SMC [52]. Either or both mechanisms may operate in vivo causing SMC to shift towards a more proliferative, synthetic phenotype in PAH. In PAD gastrocnemius, SMC with intense TGF-β1 labeling were observed frequently in the sub-endothelial region, suggesting that hypoxic insult to endothelial cells may stimulate secretion of growth factors that cause SMC to increase TGF-β1 expression. Given the histopathological similarities between PAH and PAD, future mechanistic studies of PAD myofibrosis should determine whether chronic hypoxia directly induces TGF-β1 expression by SMC, and/or activates endothelial cells to increase production of pro-fibrotic factors that cause SMC to increase expression of TGF-β1.

Understanding the mechanism by which chronic hypoxia stimulates SMC production of TGF-β1 can lead to development of animal models that recapitulate the myofibrosis observed in patients with PAD. Such models will allow for testing of novel anti-fibrotic therapies that modulate the cellular phenotypes and growth factors released from specific cell types to reduce SMC expression of TGF-β1 and myofibrosis. Importantly, we can determine side effects in these animals that may hamper the success of therapeutic strategies. Additionally, limb function can be assessed over time and in relation to changes in myofibrosis.

A limitation of this study is the number of human subjects, however our power calculations indicated enough statistical power to analyze each of the biological parameters. The main barriers against recruitment of additional human subjects are the rigorous inclusion and exclusion criteria used to reduce confounding variables, and the demands of obtaining muscle biopsies. PAD patients included in our study must have had exercise-limiting claudication established by history and direct observation during a screening walk test administered by a vascular surgeon. We enrolled only those patients who did not exhibit concurrent symptoms of heart, lung, musculoskeletal (mainly arthritis), and neurologic (mainly back pain and sciatica) ailments that would affect their walking ability and their performance in the screening walk test. Furthermore, we excluded individuals with (1) asymptomatic PAD, i.e., patients with occlusive arterial disease who do not have claudication symptoms or tissue loss/gangrene, (2) acute lower extremity ischemic events secondary to thromboembolic disease or acute trauma, and (3) exercise capacity limited by conditions other than claudication including leg (joint or musculoskeletal and neurologic) and systemic (heart and lung disease) pathology. The invasiveness of a muscle biopsy and the time required often discourages participation of PAD patients and control subjects especially when the muscle biopsy cannot be performed during their routine care (at the time of a leg operation) which was frequently the case for most PAD-II and control patients. With the 65 patients recruited, *post*-*hoc* power sample analysis using the data (mean and SD) for collagen density and area, and TGF-β1 expression, revealed adequate statistical power. Power sample analysis for an ANCOVA of TGF-β1 expression demonstrated that a total sample size of N = 65 (20, 25 and 20 subjects per group) assured at least 99 % power to detect differences between groups adjusting for two covariates with a conservative R^2^ value of 0.20. Similarly, for an ANCOVA of collagen density and area, our sample size assured at least 99 and 93 % power, respectively, to detect differences between the groups adjusting for two covariates with a conservative R^2^ value of 0.20. The power and sample size determination package PASS (PASS, Number Cruncher Statistical Systems, Kaysville, UT) was used for the analysis.

An additional limitation is the correlational nature of the study, but the high quality human data that we have presented for PAD myofibrosis provide a basis for future mechanistic studies, development of disease models, and improved therapies and prognosis. In this study, we established an association between ischemia and increased TGF-β1 production by microvascular SMC. We have identified microvascular SMC in PAD muscle as the exclusive producer of TGF-β1, making it a specific target for anti-fibrotic therapies for PAD. Candidate therapeutic drugs will be those capable of shifting the pro-fibrotic synthetic phenotype of SMC back to the contractile form to decrease TGF-β1 expression and prevent or slow down the progression of PAD myofibrosis. Moreover, TGF-β1 production by SMC may be a potential biomarker for determining the efficacy of therapeutics, including anti-fibrotic interventions. Finally, our finding of the progressive worsening of myofibrosis with advancing Fontaine Stage in PAD suggests that the patients who are optimal for anti-fibrotic intervention are those with moderate disease, since (1) a relatively small proportion of vascular SMC express TGF-β1, (2) fibroblasts are present at a relatively low density, and (3) collagen deposition is largely limited to microvessels with little expansion into the interstitium.

## Conclusions

Fibrosis is often viewed as an adaptive response to injury and tissue degeneration, but as we have shown in this study, can be part of the pathophysiology of chronic disease. We have established that increased expression of TGF-β1 by microvascular SMC in the gastrocnemius of PAD patients correlates with Fontaine Stage and increasing collagen deposition. The pattern of vascular TGF-β1 expression, fibroblast accumulation, and collagen deposition points to pathological changes in microvessels as the immediate cause of PAD myofibrosis. The contribution of hypoxia was suggested by a strong negative correlation between ABI and vascular TGF-β1 expression and presence of locally proliferative rhomboidal SMC in microvessels of PAD gastrocnemius that are indicative of the pro-fibrotic synthetic phenotype of SMC known to be induced by hypoxia. Collectively, these findings provide insight into the development of PAD myofibrosis and direction for future mechanistic studies, and consequently, a basis for improved diagnosis and treatment for patients with PAD.

## Additional file

10.1186/s12967-016-0790-3 Validation of Measurements of Collagen Deposition and TGF-β1 Expression. This supplement provides detailed methodologies and the results for validation studies of multi-spectral analysis and quantitative fluorescence microscopy and for intrasession and intersession reliability studies of collagen deposition and TGF-β1 expression measurements.

## Abbreviations

PAD: peripheral artery disease
TGF-β1: transforming growth factor beta-1
SMC: smooth muscle cells
ABI: ankle brachial index
