# Supplementary material for: Transforming growth factor-beta 1 produced by vascular smooth muscle cells predicts fibrosis in the gastrocnemius of patients with peripheral artery disease
Source: J Transl Med. 2016 Feb 4;14:39. doi: 10.1186/s12967-016-0790-3 (PMC4743093; doi:10.1186/s12967-016-0790-3)
Supplement: Supplementary file 1 — 10.1186/s12967-016-0790-3 Validation of Measurements of Collagen Deposition and TGF-β1 Expression. This supplement provides detailed methodologies and the results for validation studies of multi-spectral analysis and quantitative fluorescence microscopy and for intrasession and intersession reliability studies of collagen deposition and TGF-β1 expression measurements. [file 12967_2016_790_MOESM1_ESM.pdf]

VALIDATION OF MEASUREMENTS OF COLLAGEN DEPOSITION AND TGF-β1 EXPRESSION

Validation of Spectral Analysis: Spectral analysis was validated by the hydroxyproline assay for collagen content in whole muscle. A robust positive correlation **(r = 0.907; p < 0.001)** was detected between hydroxyproline (μg per mg muscle) and optical density from spectral images, for 10 patients who encompassed a wide range of collagen spectral densities **(Figure S1A)**.

Hydroxyproline Assay: Total collagen content of human gastrocnemius specimens was measured by the hydroxyproline assay. Four PAD-IV, three PAD-II, and three CTRL subjects were selected for analysis based on their wide range of collagen density determined by wide-field multi-spectral microscopy. Briefly, approximately 10 mg of liquid nitrogen preserved gastrocnemius muscle from each patient was hydrolyzed in hydrochloric acid (Fisher Scientific #SA56-1, Pittsburg, PA, USA) overnight for 12 hours at 130° C. Then, contents were adjusted to neutral pH by potassium hydroxide (Sigma #C9887, St Louis, MO, USA) using a solution of phenolphthalein (Sigma #34607, St Louis, MO, USA) as indicator, and buffered with Sodium Borate that is composed of Sodium Hydroxide (Fisher Scientific #S318-500, Pittsburg, PA, USA) and Boric Acid (Fisher Scientific #BP168-500, Pittsburg, PA, USA). Hydroxyproline was oxidized by the Chloramine T solution, which is comprised of chloramine T (Sigma #C9887, St Louis, MO, USA), citric acid anhydrous (Sigma #C0759, St Louis, MO, USA), glacial acetic acid (Fisher Scientific #A38-212, Pittsburg, PA, USA), sodium acetate trihydrate (Sigma #S7670, St Louis, MO, USA), sodium hydroxide (Fisher Scientific #S318-500, Pittsburg, PA, USA), and 2-mthoxyethanol (Sigma #284467, St Louis, MO, USA). Then, the reaction was stopped by Sodium Thiosulfate (Sigma #217263, St Louis, MO, USA). Impurities were removed by addition of toluene (Sigma #244511, St Louis, MO, USA), inversion and centrifugation, and aspiration of the resulting toluene layer. Next, the oxidized product was extracted by inversion, centrifugation, and collection of the toluene layer. Finally, Ehrlich’s Reagent, a mixture of sulfuric acid (Sigma #339471, St Louis, MO, USA), ethanol (Decon Labs #2701, King of Prussia, PA, USA), and 4-dimethylaminobenzaldehyde (Sigma #156477, St Louis, MO, USA) was added to the extraction to

initiate a colorimetric reaction and incubated at room temperature for 30 minutes. These samples and hydroxyproline standards (0.0 - 8.0 µg; Sigma #H54409, St Louis, MO, USA) were analyzed at 560-nm wavelength on a spectrophotometer. The hydroxyproline content of muscle was expressed as µg per mg muscle (wet weight).

Reproducibility of Spectral Analysis: To determine intersession reliability, slide specimens of biopsy samples from 10 patients were stained by Masson's Trichrome in two separate analytical sessions and assessed by spectral analysis. The Intraclass Correlation Coefficient (ICC) was estimated by using a two-way mixed ANOVA with absolute agreement. An ICC value of less than 0.40 indicates poor reproducibility, between 0.40 and 0.75 indicates fair to good reproducibility, and greater than 0.75 indicates excellent reproducibility. The ICC (0.865) indicated an excellent reproducibility **(Figure S1B)** with an average difference from the mean of the two analytical sessions of 4%.

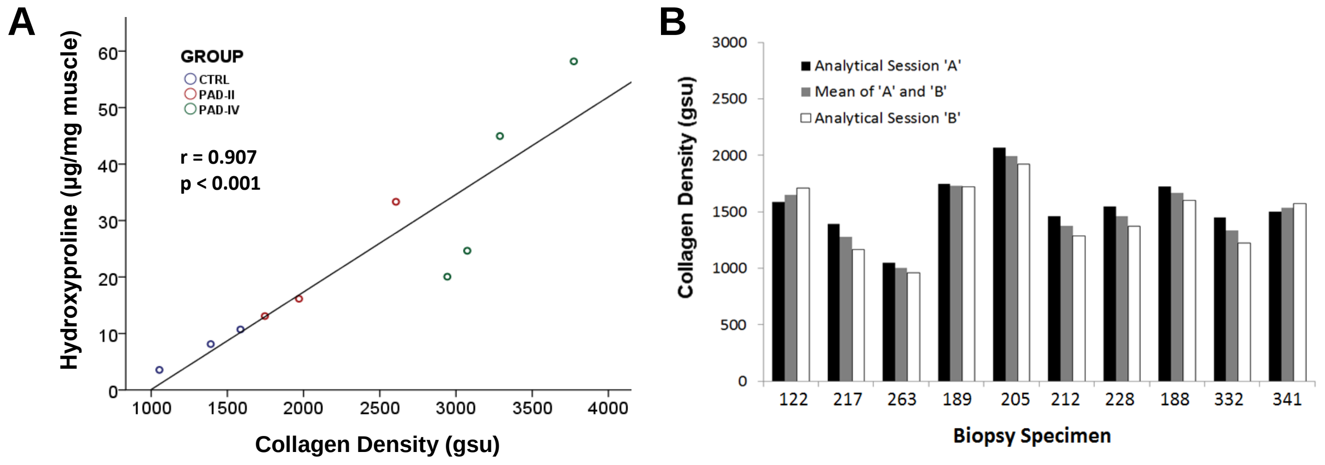

Figure S1. Validation of Multi-spectral Microscopic Analysis of Collagen Deposition. (A) Pearson correlation demonstrating the relationship between collagen density measurements by multi-spectral microscopic analysis and hydroxyproline content in muscle homogenate, as measured by the hydroxyproline assay. **(B)** Reliability of multi-spectral measurements of collagen density in independent, analytical sessions A and B was determined as an intraclass correlation (ICC) value of 0.865, representing excellent reliability (ICC > 0.75 is excellent, 0.75 ≥ ICC ≥ 0.40 is good, and ICC < 0.40 is poor).

Validation of Quantitative Fluorescence Microscopy: QFM measurements of TGF-β1 were validated by comparing results with ELISA and qPCR measurements of muscle homogenates from PAD-II and CTRL patients (N=13 in each group). Additionally, the TGF-β1 expression pattern in PAD and control gastrocnemius was confirmed with a second anti-TGF-β1 antibody (Abcam Ab170736, Cambridge, MA, USA).

ELISA: Conventional sandwich-based ELISA measurements of TGF-β1 expression in PAD gastrocnemius was carried out according to manufacturer instructions as part of a customized Human Inflammatory Cytokines Multi-Analyte ELISArray Kit (Qiagen, Valencia, CA, USA). TGF-β1 expression by QFM demonstrated significant positive correlations with TGF-β1 determined by ELISA **($r = 0.602$; $p = 0.001$; Figure S2A)**.

Quantitative PCR: TGF-β1 RNA transcripts in skeletal muscle biopsies stored in liquid nitrogen were extracted, reverse transcribed, quantified by qPCR, and normalized to myosin gene transcripts. The following TGF-β1 primers (188 bp, NCBI, NM_000660) were used: forward, 5'-CCGCAAAGACTTTTCCCCAGACC-3'; reverse, 5'- ACCTAGATGGGCGCGATCTGGTA-3'. The following myosin primers (199 bp; NCBI, NM_005963) were used: forward, 5'-TCCGAAAGTCTGAAAGGGAGCGAA-3'; reverse, 5'-GAGGGTTCATGGGGAAGACTTGGT-3'. TGF-β1 expression by QFM demonstrated significant positive correlations with TGF-β1 transcripts as determined by qPCR **($r = 0.659$; $p < 0.001$; Figure S2B).**

Reliability of Quantitative Fluorescence Microscopy: The intersession reliability was determined for TGF-β1 from the averages of patient biopsy specimens, analyzed a second time in the next analytical session. The two-session mean of each biopsy specimen was determined and each session mean was expressed as the absolute difference from the two-session mean. The ICC was estimated by a two-way mixed ANOVA with absolute agreement. The ICC (0.993) indicated excellent reproducibility **(Figure S2C)**, with an average absolute difference from the mean of the two analytical sessions of 5.3%.

Overall, QFM is a reliable method to measure expression of TGF-β1 in skeletal muscle biopsy specimens. The intrasession reliability was high (ICC = 0.957), with a 6% average absolute difference of each slide from the mean of its duplicate pair.

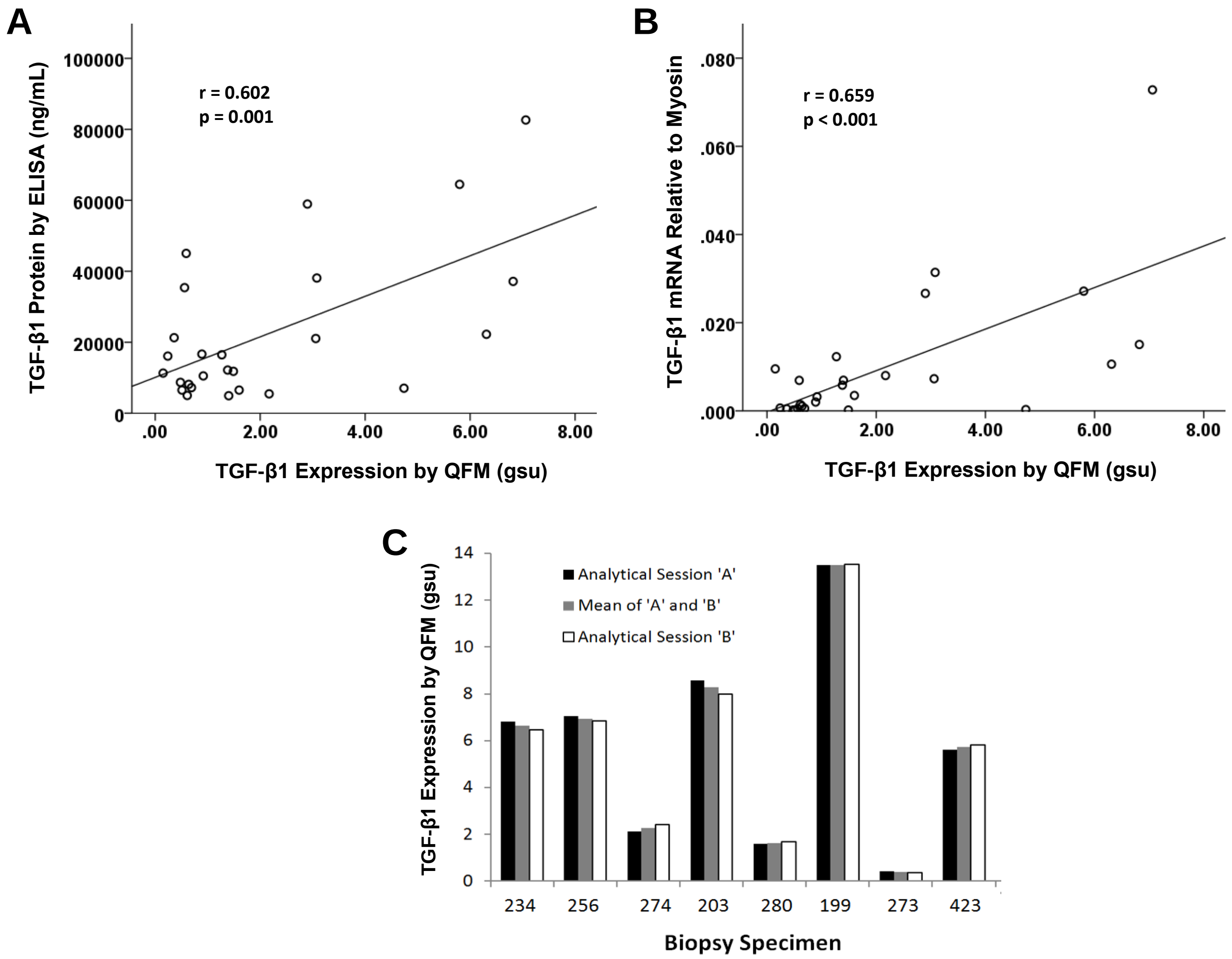

Figure S2. Validation of Quantitative Fluorescence Microscopic (QFM) Analysis of TGF-β1. The relationships between TGF-β1 expression determined by QFM and TGF-β1 expression determined by **(A)** ELISA, and **(B)** qPCR in 13 controls and 13 PAD-II patients were analyzed by Pearson correlation. **(C)** Intersession reliability of normalized TGF-β1 values obtained with slides from the same patient specimens in two independent analytical sessions A and B was determined as an intraclass correlation (ICC) value of 0.918, representing excellent reliability (ICC > 0.75 is excellent, 0.75 ≥ ICC ≥ 0.40 is good, and ICC < 0.40 is poor).
